# Downregulation of SHIP2 by Hepatitis B Virus X Promotes the Metastasis and Chemoresistance of Hepatocellular Carcinoma through SKP2

**DOI:** 10.3390/cancers11081065

**Published:** 2019-07-27

**Authors:** Kuo-Jung Su, Yung-Luen Yu

**Affiliations:** 1 The Ph.D. Program for Cancer Biology and Drug Discovery, China Medical University and Academia Sinica, Taichung 404, Taiwan; 2 Graduate Institute of Biomedical Sciences, China Medical University, Taichung 404, Taiwan; 3 Center for Molecular Medicine, China Medical University Hospital, Taichung 404, Taiwan; 4 Drug Development Center, China Medical University, Taichung 404, Taiwan; 5 Department of Biotechnology, Asia University, Taichung 413, Taiwan

**Keywords:** SHIP2, SKP2, HBx, hepatocellular carcinoma (HCC), migration

## Abstract

Hepatitis B virus (HBV)-encoded X protein (HBx) plays an important role in the development of hepatocellular carcinoma (HCC). The protein SH2 domain containing inositol 5-phosphatase 2 (SHIP2) belongs to the family of enzymes that dephosphorylate the 5 position of PI(3,4,5)P3 to produce PI(3,4)P2. Expression of SHIP2 has been associated with several cancers including HCC. However, its role in the development of HBV-related HCC remains elusive. In this study, we performed tissue microarray analysis using 49 cases of HCC to explore SHIP2 expression changes and found that SHIP2 was downregulated in HBV-positive HCC. In addition, S-phase kinase-associated protein 2 (SKP2), a component of the E3 ubiquitin–ligase complex, was increased in HCC cell lines that overexpressed HBx, which also showed a notable accumulation of polyubiquitinated SHIP2. Moreover, HCC cells with silenced SHIP2 had increased expression of mesenchymal markers, which promotes cell migration, enhances glucose uptake, and leads to resistance to the chemotherapy drug (5-Fluorouracil, 5-FU). Taken together, our results demonstrate that HBx downregulates SHIP2 through SKP2 and suggest a potential role for SHIP2 in HBx-mediated HCC migration.

## 1. Introduction

Hepatocellular carcinoma (HCC) is one of the most common malignancies and is a leading cause of cancer-related deaths in the world. Globally, the frequency of hepatitis B surface antigen in the general population ranges from 2% to 20%, indicating chronic infection with hepatitis B virus (HBV), which leads to the development of HCC [1,2]. HBV x protein (HBx), which is encoded by the smallest open reading frame of the HBV genome, is associated with hepatocellular carcinogenesis [3,4]. Furthermore, HBx is a multifunctional viral regulator that modulates protein degradation via interacting with proteasome subunits [5,6]. Although, HBx is strongly associated with the development of HCC, the underlying molecular mechanisms for this association remain unknown.

The ubiquitin–proteasome system has an important role in the regulation of diverse cellular processes, including cell cycle control, transcription, apoptosis, cell adhesion, and tumor growth, through targeted degradation of regulatory proteins [7,8]. The S-phase kinase-associated protein 2 (SKP2) F-box protein is a component of the SCF type of E3 ubiquitin–ligase complex that recruits target proteins for ubiquitination [9]. For example, SKP2 acts as the substrate recognition factor by binding to and targeting the tumor suppressor p27 for degradation [10]. In addition, mutations in HBx can lead to the downregulation of expression of p21 via the SKP2-mediated proteasomal degradation pathway, which might increase the risk of HCC [11]. Overexpression of SKP2 is frequently observed in cancer progression and metastasis [12], and recent studies have demonstrated that SKP2 behaves as an oncogene [13]. However, the molecular mechanism of oncogenic cooperation between HBx and SKP2 is still not clear.

Phosphatidylinositol 3,4,5-triphosphate (PI(3,4,5)P3) is a critical signaling component that has major regulatory roles in a variety of cellular functions [14]. SHIP2 belongs to the family of mammalian inositol polyphosphate 5-phosphatases, which convert PI(3,4,5)P3 into PI(3,4)P2, a negative regulator of phosphoinositide 3-kinase (PI3K) and insulin signaling [15,16,17]. SHIP2 contains an N-terminal SH2 domain, a catalytic domain, potential phosphotyrosine binding (PTB) domain–binding sites (NPXY), C-terminal proline-rich regions, and a sterile α motif (SAM) [18]. Moreover, a ubiquitin-interacting motif at the C-terminal end of SHIP2 enables the protein to bind ubiquitin [19]. SHIP2 knockout in mice produces an increase in glycolysis and glycogen storage with reduced gluconeogenesis [20]. Cancer cells activate glycolysis for ATP generation irrespective of the presence or absence of oxygen (the Warburg effect), which increases cancer cell proliferation and survival [21]. In addition, a higher glycolytic rate in cancer cells increases drug resistance by supporting uninterrupted growth [22]. Therefore, inhibition of glycolysis in cancer cells is a novel strategy to effectively kill cancer cells and overcome drug resistance. However, SHIP2 has been reported to have both tumor growth and tumor suppression roles depending on the cell model. In breast cancer, SHIP2 regulates epidermal growth factor receptor (EGFR) levels to support cell proliferation and tumor growth [14]. In contrast, SHIP2 causes cell cycle arrest in glioblastoma, and its expression is downregulated in HCC patients [23,24]. SHIP2 is also linked to the regulation of the actin cytoskeleton, cell adhesion, and migration. However, no definitive role for SHIP2 in the regulation of HCC has been established.

In this study, we examined the expression of SHIP2 in HCC cell lines and in HBV-infected HCC tissues. We found that HBx induced downregulation of SHIP2 in HBV-HCC. Additionally, downregulation of SHIP2 in HCC was correlated with increased migration and invasion. Furthermore, we demonstrated that SKP2 is able to directly regulate SHIP2 in the promotion of HCC migration mediated by HBx. To our knowledge, this is the first study that addressed the effect of HBx on SHIP2 regulation. This study provides evidence to support SHIP2 as a novel approach to target HBV-related HCC.

## 2. Results

### 2.1. SHIP2 Is Downregulated in HBV-Infected HCC Tissues

**Figure 1:**
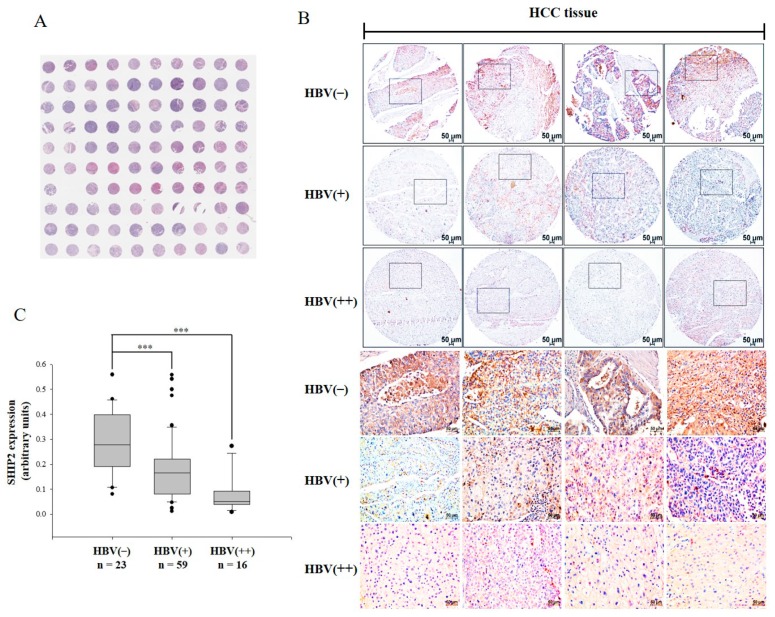
SH2 domain containing inositol 5-phosphatase 2 (SHIP2) is downregulated in human hepatocellular carcinoma (HCC) tissue with hepatitis B virus (HBV) infection. (**A**) Representative hematoxylin and eosin (H&E)-stained tissue microarray (TMA) slide, containing 98 duplicate core tissue samples from 49 individuals with HCC. Scale bar: 50 μm. (**B**) Expression of SHIP2 in human HCC tissue without or with HBV infection was examined by immunohistochemistry (IHC) analysis of the TMA, which was stained with human SHIP2 antibody. Representative cores from the human HCC TMA are shown, and the areas within the black squares are enlarged and presented in the lower panels. (**C**) Quantification of IHC intensity for SHIP2 in cores grouped according to HBV infection status. Data represent the mean ± standard deviation (s.d.), *** *p* < 0.001.

Table 1 summarizes the characteristics of the individuals from whom the liver cancer tissue microarray (TMA) was constructed, including analysis for HBV infection. The TMA includes 49 cases of hepatocellular carcinoma and 1 case of mixed hepatocellular carcinoma and cholangiocellular carcinoma (excluded here), of which duplicate HCC tissue samples were analyzed for each case. The TMA slide included 42 males and 7 females with HCC, ranging in age from 24 to 68 years (median age, 50 years). The tumors were classified as stage I in 5 patients, stage II in 42 patients, and stage III in 2 patients. The level of HBV infection was also classified as “− (no staining)” in 23 cores, “+ (weak staining)” in 59 cores, and “++ (moderate staining)” in 16 cores. A representative hematoxylin and eosin (H&E)-stained TMA slide is shown in Figure 1A.

To investigate the role of SHIP2 in HCC, we first evaluated the expression of SHIP2 in all 49 cases with or without HBV infection by TMA and immunohistochemistry (IHC). Expression of SHIP2 was notably lower in the HBV-infected HCC tissues than in those without HBV infection (Figure 1B and Figure S1). Quantification of IHC intensity confirmed that SHIP2 expression was significantly decreased in HBV-infected HCC tissues (*p* < 0.001) (Figure 1C). These data suggested that HBV might reduce SHIP2 expression in HBV-infected patients with HCC.

### 2.2. HBx Reduces SHIP2 Expression in HCC Cells

**Figure 2:**
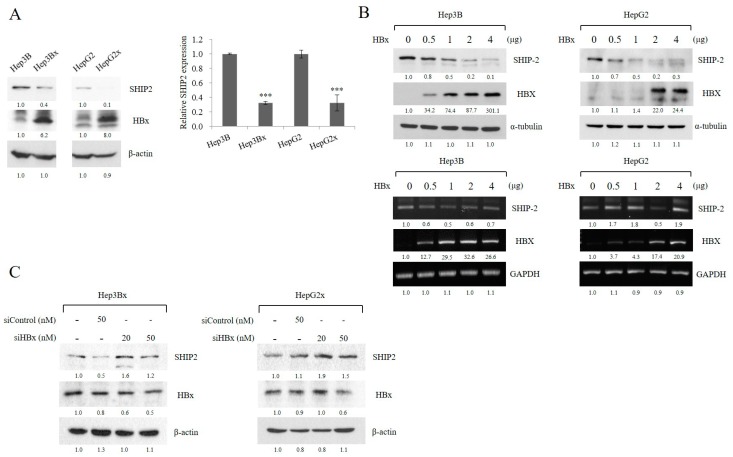
Hepatitis B virus (HBV)–encoded X protein (HBx) reduces SHIP2 protein expression in HCC cells. (**A**) SHIP2 was reduced in HBx-expressing Hep3Bx and HepG2x HCC cell lines, as compared with parental lines. Quantification of relative SHIP2 expression is shown (*n* = 3). Data represent the mean ± s.d., *** *p* < 0.001. (**B**) HBx expression vector was transiently transfected into Hep3B and HepG2 cell lines for 48 h. SHIP2 and HBx expression was analyzed at the protein level by western blotting and at the mRNA level by RT-PCR. (**C**) Transient transfection of HBx and control siRNA was performed in Hep3Bx and HepG2x cells for three days. SHIP2 expression and gene silencing of HBx protein expression were examined by western blotting. In all the above cases, α-tubulin, GAPDH (Glyceraldehyde 3-phosphate dehydrogenase), and β-actin are used as the internal control. The whole blot has been provided as Supplemental Materials (Figure S4).

To assess whether expression of SHIP2 is responsive to HBx, we first examined the expression of SHIP2 in HCC cell lines (Hep3B and HepG2, which do not express HBx) and in their derivatives that stably express HBx (Hep3Bx and HepG2x). Consistent with the previous observation in TMA, SHIP2 expression was significantly lower in HBx-expressing cells than in the parental cells (Figure 2A). To further determine the relationship between HBx and SHIP2, the effect of HBx overexpression and gene silencing was examined. We found that transient transfection of the HBx plasmid into Hep3B and HepG2 cells resulted in a reduction in endogenous SHIP2 protein expression in a concentration-dependent manner (Figure 2B; top). This result is consistent with SHIP2 expression in cell lines that stably express HBx. However, HBx did not significantly affect the mRNA level of SHIP2 (Figure 2B; bottom). We thus hypothesized that HBx might reduce SHIP2 expression via the ubiquitin–proteasome pathway. In contrast, expression of endogenous SHIP2 in Hep3Bx and HepG2x cells was increased when those cells were transfected with HBx short-interfering RNA (siRNA) (Figure 2C). Taken together, these results suggest that HBx expression is responsible for the reduction in SHIP2 protein levels in HCC cells.

### 2.3. SHIP2 Is Regulated by HBx through SKP2 in HCC Cells

**Figure 3:**
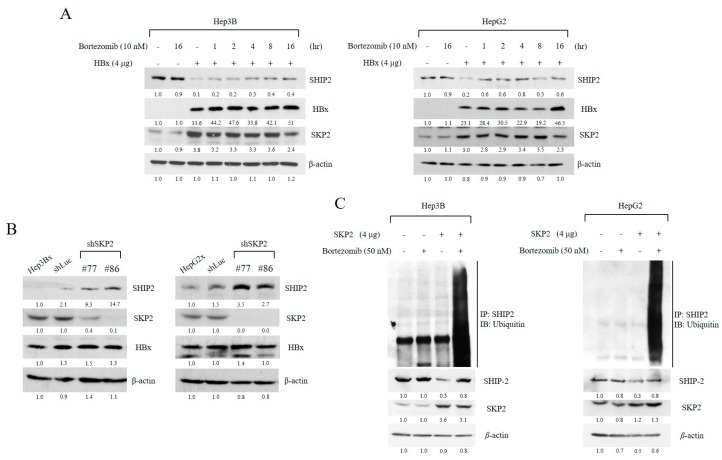
SHIP2 is polyubiquitinated by the E3 ubiquitin ligase SKP2 in HCC cells. (**A**) Time-dependent study of the effect of bortezomib (10 nM) treatment on SHIP2 expression in HBx-transfected HCC cell lines. SHIP2, HBx, and S-phase kinase-associated protein 2 (SKP2) were analyzed by western blotting. (**B**) Hep3Bx and HepG2x cells were infected with lentivirus for delivery of short-hairpin RNA (shRNA) against luciferase (shLuc) or SKP2 (shSKP2) for three days, and then whole-protein lysates were analyzed by western blotting. (**C**) Hep3B and HepG2 cells were transiently transfected with SKP2 or control expression vector for 48 h and then were treated in the absence or presence of bortezomib (50 nM) for 16 h prior to cell harvest. Whole-protein lysates were prepared followed by immunoprecipitation (IP) with anti-SHIP2. The precipitate was then electrophoresed on a polyacrylamide gel followed by immunoblotting (IB) using anti-ubiquitin. The whole blot has been provided as Supplemental Materials (Figure S5).

We next determined whether endogenous SHIP2 is modified by ubiquitin. As the ubiquitin E3 ligase SKP2 interacts with HBx [25], we determined whether SKP2 might be involved in SHIP2 degradation. First, Hep3B and HepG2 cells were transiently transfected with HBx plasmid or a control plasmid and then were treated with proteasome inhibitor (bortezomib) for various times. Treatment of Hep3B and HepG2 with bortezomib caused a time-dependent increase in SHIP2 levels (Figure 3A). A notable increase in SHIP2 protein was detected at 1 h after bortezomib treatment. We also observed a substantial increase in SKP2 protein after transfection with HBx plasmid. These results indicated that SKP2 in particular may contribute to the decrease in SHIP2 expression in the presence of HBx.

To provide direct evidence whether upregulation of SKP2 downregulates SHIP2, we attempted to inhibit SKP2 expression in HBx-expressing cell lines by short-hairpin RNA (shRNA). SKP2 protein levels were greatly reduced in cells infected with shSKP2 for 72 h (Figure 3B). Furthermore, we observed that depletion of SKP2 led to increased SHIP2 expression, indicating that SKP2 is involved in the regulation of SHIP2 levels.

To further implicate the involvement of the proteasome pathway, the levels of ubiquitinated SHIP2 were assessed in HCC cells after transfection with SKP2 plasmid. In Hep3B and HepG2, SKP2 induced a notable accumulation of polyubiquitinated SHIP2 after treatment with bortezomib (Figure 3C). Moreover, we also observed the identical results after cotransfecting SHIP2 and SKP2 into HEK293 cells (Figures S2 and S7). Taken together, our results indicated that SHIP2 expression was downregulated by the ubiquitin ligase SKP2 as a result of HBx expression.

SHIP2 is involved in the regulation of cell motility through the epithelial–mesenchymal transition (EMT) in HCC cells 

**Figure 4:**
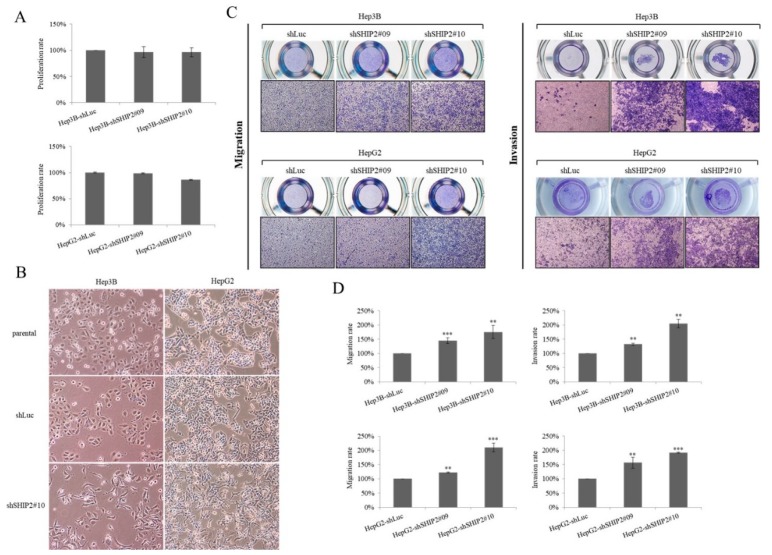
Inhibition of SHIP2 enhances cell migration and invasion in HCC cell lines. (**A**) The proliferation ability of Hep3B and HepG2 cells grown in 96-well plates was assessed using a WST-1 assay for a period of 24 h after SHIP2 shRNA knockdown. (**B**) Representative images of cell morphology in parental, shLuc, and shSHIP2 cells. Magnification, 100×. (**C**) Cell migration and invasion were measured using Transwell assays for shLuc, and shSHIP2 cells. Representative photographs from at least three different experiments are shown. Magnification, 100× (**D**) The relative migration and invasion rates of cells as shown in (C) were determined by crystal violet staining and quantification. Statistical analysis was performed with a Student’s *t*-test. ** *p* < 0.01; *** *p* < 0.001 as compared with the shLuc group (*n* = 3).

To further understand the mechanism of HBx-promoted migration, we examined the effect of SHIP2 on the migration ability of HCC cells by knockdown of SHIP2 expression. Knockdown of SHIP2 expression did not significantly affect cell proliferation in Hep3B and HepG2 cells (Figure 4A). However, compared with parental cells, SHIP2 knockdown in HCC cells revealed morphological changes, with a more scattered and spindle-shaped appearance, indicating an increase in the mobility of HCC cells (Figure 4B). We next investigated the functional roles of decreased SHIP2 expression. The results showed that inhibition of SHIP2 promoted the migration and invasion of HCC cells as compared with the control (Figure 4C,D). These results clearly indicated that SHIP2 regulates cell motility in the HCC cell lines.

**Figure 5:**
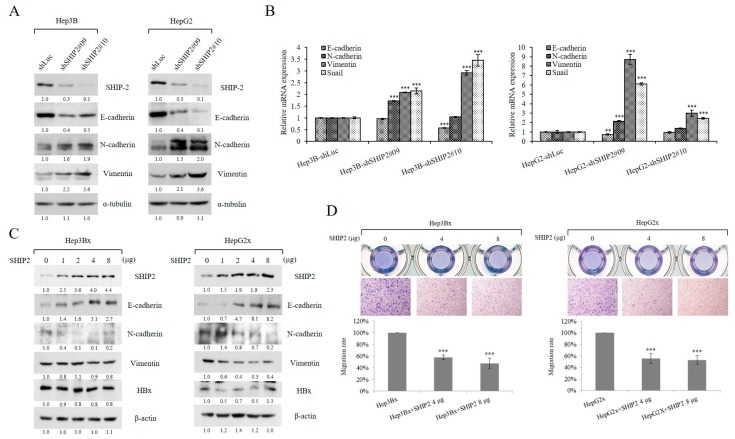
SHIP2 regulates cell motility through the epithelial–mesenchymal transition (EMT) in HCC cell lines. (**A**) An epithelial marker (E-cadherin), mesenchymal markers (N-cadherin and vimentin), and SHIP2 were detected by western blotting in control and SHIP2-knockdown HCC cell lines. (**B**) The mRNA expression of EMT markers was examined by real-time PCR in control and SHIP2-knockdown HCC cell lines. mRNA expression was normalized to actin expression. (**C**) Hep3Bx and HepG2x cells were transiently transfected with SHIP2 plasmid for 48 h. The SHIP2 and EMT markers were examined by western blotting. (**D**) The HBx-expressing HCC cell lines were transfected with SHIP2 plasmid for 48 h. The migration rate was then examined by the Transwell migration assay after a 24 h incubation. Magnification, 100×. Representative images of migrated cells are shown. Statistical analysis was performed by Student’s *t*-test. ** *p* < 0.01; *** *p* < 0.001 as compared with the control group (*n* = 3). The whole blot has been provided as Supplemental Materials (Figure S6).

The EMT contributes to the cell migration of different cancers [26]. We further tested the importance of SHIP2 in influencing EMT characteristics in HCC cells. We found that knockdown of SHIP2 expression increased the relative expression of mesenchymal markers (N-cadherin, vimentin, and snail) but decreased an epithelial marker (E-cadherin), as demonstrated by immunoblotting and real-time PCR (Figure 5A,B). However, this effect was reversed when SHIP2 was overexpressed in Hep3Bx and HepG2x cells (Figure 5C). In addition, overexpression of SHIP2 was able to abolish the enhanced migration of HBx-expressing HCC cells mediated by HBx (Figure 5D). Taken together, these data suggested that SHIP2 might contribute to the regulation of HCC cell motility thought EMT. 

### 2.4. Downregulation of SHIP2 Leads to Elevated Glucose Uptake and Drug Resistance to 5-FU

**Figure 6:**
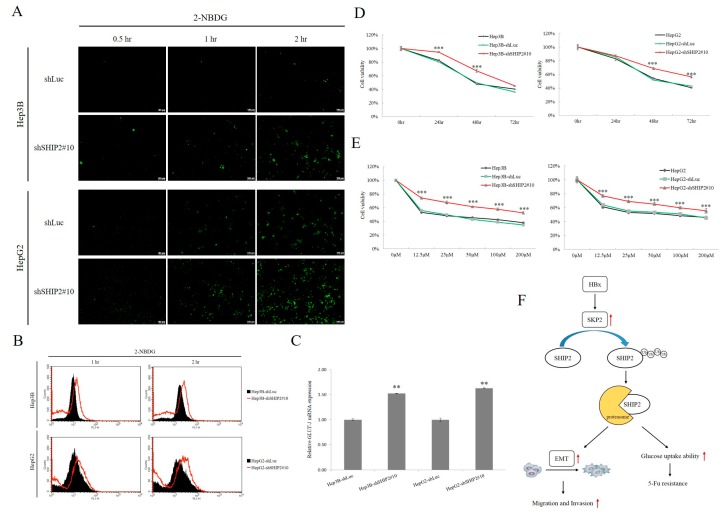
Inhibition of SHIP2 activates glucose uptake and 5-FU resistance in HCC cells. (**A**,**B**) Time-dependent glucose uptake in HCC cells following SHIP2 knockdown. Glucose uptake was analyzed with the fluorescent deoxyglucose analog 2-NBDG by fluorescence microscopy and flow cytometry. Tumor cells were incubated with 50 nM 2-NBDG for 0.5, 1, and 2 h. The lentivirus containing shRNA against luciferase (shLuc) was used as the control. Representative data from three independent experiments are shown. (**C**) *GLUT1* mRNA expression in SHIP2-knockdown HCC cell lines was examined by real-time PCR. *GLUT1* mRNA expression was normalized to actin expression. ** *p* < 0.01 (**D**,**E**) The cytotoxic effect of 5-FU in SHIP2-knockdown HCC cells was analyzed. Hep3B and HepG2 cells were plated in 96-well plates after shLuc or SHIP2 knockdown for 72 h. The parental lines were also plated as additional controls. The cells were treated with the indicated doses (12.5–200 μM) of 5-FU for 48 h or were treated with a time course of 25 μM 5-FU for 24, 48, and 72 h. After 5-FU treatment, cell survival was monitored using the WST-1 assay. Statistical analysis was carried out with Student’s *t*-test. *** *p* < 0.001 as compared with each control group (*n* = 3). (**F**) Schematic diagram depicting how HBx mediates SHIP2 in HCC cells.

Cancer cells exhibit increased glycolysis even in the presence of oxygen, which is now known as the Warburg effect [21]. To test whether glycolysis is affected by SHIP2 inhibition, we analyzed glucose uptake and the expression of glucose transporter 1 (GLUT1) in the HCC cells. The fluorescently labeled deoxyglucose analog 2-NBDG (2-(*N*-(7-nitrobenz-2-oxa-1,3-diazol-4-yl)amino)-2-deoxyglucose) is used to directly monitor glucose uptake by living cells and tissues [27]. SHIP2 knockdown with shRNA in HCC cells led to a notable time-dependent increase in the intracellular uptake of glucose, as based on 2-NBDG fluorescence (Figure 6A,B). To elucidate a possible mechanism behind the activation of glucose uptake after SHIP2 inhibition, we investigated the potential involvement of glucose transporter molecules such as GLUT1. We observed that knockdown of SHIP2 significantly increased *GLUT1* mRNA expression in HCC cells (Figure 6C). As previous studies demonstrated that dysregulation of glucose metabolism is linked to chemoresistance in cancer cells, we determined whether SHIP2 inhibition in HCC cells may affect the sensitivity of these cells to chemotherapy drugs. We exposed the SHIP2-knockdown cells to various concentrations of chemotherapeutic agents, such as Iressa, Tarceva, Doxorubicin, Sorafenib, Paclitaxel, and 5-FU, and monitored their viability (Figure S3). Interestingly, we observed a significant resistance only to 5-FU treatments at multiple concentrations and over multiple time points. A 25 μM dose of 5-FU significantly inhibited Hep3B cell viability to 50% within 48 h, whereas a concentration of as much as 200 μM more was required to inhibit viability of the SHIP2-knockdown cells (Figure 6D,E). 

## 3. Discussion

PI3K has a crucial role in cellular functions such as cell growth, proliferation, differentiation, motility, and survival, producing as a result the phosphoinositides (phosphatidylinositol phosphates) PtdIns(3)P1, PtdIns(3,4)P2, and PtdIns(3,4,5)P3 [28]. A recent report demonstrated that PTEN and SHIP2, which are phosphatases that widely act as negative regulators of insulin signaling, are responsible for dephosphorylation of PtdIns(3,4,5)P3 [29]. PTEN is one of the most common tumor suppressors, and mutations in or deletions of PTEN are frequently found in various human cancers and promote tumorigenesis [30]. However, the effects of SHIP2 on the regulation of tumor development have yet to be fully elucidated, prompting our investigation into whether SHIP2 inhibition affects the malignant intracellular processes of HBx in HCC. 

SHIP2 is an important regulator of energy metabolism, such as its negative regulation of insulin signaling and its correlation with susceptibility to type 2 diabetes [31]. SHIP2 has both tumorigenic and tumor suppressor functions [32]. In glioblastoma cells, SHIP2 overexpression leads to a potent cell cycle arrest in the G1 phase, suggesting a negative effect of SHIP2 on cell proliferation. In addition, SHIP2 negatively regulates cell substrate adhesion and migration in normal human keratinocytes [33]. However, SHIP2 also positively regulates cell adhesion and migration via interactions with cytoskeletal regulators, and its expression is correlated with poor prognosis in breast cancer cells [34]. Taken together, these results suggest that the role of SHIP2 in cancer cells is cell-type specific. Therefore, we hypothesized that there might be a strong interactive link between SHIP2 and EMT regulators in HCC. Indeed, we found that deletion of SHIP2 induced the in vitro migration, invasion, and expression of mesenchymal markers (N-cadherin and vimentin). Moreover, HBx has been found to repress E-cadherin, which is important for the maintenance of cell polarity in tissue [35]. Thus, further investigations are important to reveal the link between SHIP2 and other EMT regulators. Interestingly, Awad et al. reported that HCV (Hepatitis C virus) core protein disrupted cell polarity and infected polarized cells, accompanied by a significant reduction of SHIP2 expression and its lipid product, PI(3,4)P2 [36]. This study confirmed that the role of PI(3,4)P2 and SHIP2 are critical to maintain apico–basal polarity. The invasive HCC typically contains cells that escaped from their original context and lost apical–basal polarity to promote development of carcinogenesis. Disturbed polarization is a hallmark of cancer, and thus HCV-induced loss of polarity mechanisms dependent on downregulation of SHIP2 promote invasion of cells and the development of carcinogenesis. In our study, TMA data showed that tumor tissues contained lower SHIP2 levels associated with higher HBx levels than adjacent normal tissues. Taken together, these results provide a possible molecular mechanism that may explain how HBV infection worsens the clinical outcome in HCC patients.

Recent studies have been published regarding associations between HBV and HCC development. The HBV genome has four overlapping open reading frames (ORFs), which code for the viral envelope, core proteins, viral polymerase, and HBx protein. Among the four proteins that originate from the HBV genome, the HBx protein plays an important role in multiple steps of HCC development [37]. HBx also regulates a few E3 ligases, such as SKP2 F-box protein, which is the recruiting component of the SCF type of E3 ubiquitin–ligase complex. SKP2 targets many proteins for subsequent degradation and ubiquitination, including the tumor suppressors p21, p27, and p57 [38]. SKP2 overexpression is frequently found in human cancer and plays a critical role in tumorigenesis. Although SKP2 is an important regulator that has been widely studied in various cancer types, the role of SKP2 in HBV-infected HCC has rarely been analyzed. Mutations in the core HBV promoter in combination with HBx mutations can upregulate SKP2, which then downregulates tumor suppressors p21 and p53 via ubiquitin-mediated proteasomal degradation [11,39]. We found that the level of *SHIP2* mRNA was not affected by HBx expression. Therefore, we hypothesize that HBx modulates SHIP2 expression through SKP2 and the ubiquitin–proteasome pathway. Our results clearly indicated that HBx induced SKP2 expression and thereby induced a notable accumulation of polyubiquitinated SHIP2. Taken together, our results suggest that HBx mediates migration and invasion of HCC cells by SHIP2 downregulation and stimulation of its degradation by the E3 ligase SKP2.

The Warburg effect is a hallmark of malignant cancers, which show elevated uptake of glucose even under normal oxygen conditions. Overexpression of SHIP2 results in the inhibition of glucose uptake in L6 myotubes [40]. In addition, expression of SHIP2 inhibits insulin stimulation of glucose transport via the 5′-phosphatase activity in 3T3-L1 adipocytes [41]. However, the ability of SHIP2 to regulate glucose metabolism in HCC cells remains unclear. Here we demonstrated that SHIP2 inhibition could enhance glucose uptake through activation of GLUT1 expression in HCC cells. Importantly, the high rate of glucose uptake induces drug resistance in cancer cells [42]. In the present study, we found that an increase in glucose uptake ability as a result of SHIP2 inhibition, which is closely associated with HBV infection in HCC cells, may increase the resistance of these cells to 5-FU (Figure 6F). Our findings, together with those from previous studies, underscore the important role of SHIP2 as a molecular regulator linking glucose metabolism and 5-FU resistance in HBV infection. Further research is required to survey the importance of SHIP2 in HBV infected patients, and the relation with the other determinants of SKP2, metastasis and HCC.

## 4. Materials and Methods

### 4.1. TMA Analysis

Correlation of SHIP2 expression with HBV infection of hepatocellular carcinoma liver tissue was examined using the established TMA LV1003 (US Biomax, Rockville, MD, USA). This microarray includes 49 cases of hepatocellular carcinoma and 1 case of mixed hepatocellular carcinoma and cholangiocellular carcinoma (excluded here) with TNM classification and pathology grades of the tumors. Standard IHC staining procedures (Rapid Science Co., Ltd., Taichung, Taiwan) were performed using anti-SHIP2 (Santa Cruz Biotechnology, Dallas, TX, USA). The staining results were scored by two investigators blinded to the clinical data. Briefly, stained tissue sections were viewed under a light microscope, and images were captured by DS-Fi1 digital microscope camera head (Nikon, Melville, NY, USA). To determine the intensity of SHIP2 expression, images were analyzed using ImageJ software (National Institutes of Health, Bethesda, MD, USA) as described [43]. Briefly, IHC intensity was quantified by measuring pixel intensities by an investigator without any information regarding the group assignment of the tissue. 

### 4.2. Cell Culture

Hep3B and HepG2 cell lines (both were from ATCC) were cultured in Dulbecco’s modified Eagle’s medium (DMEM)-F12 medium supplemented with 10% fetal bovine serum (Invitrogen, Carlsbad, CA, USA), 100 U/mL penicillin and 100 μg/mL streptomycin (Thermo, Waltham, MA, USA). HepG2x and Hep3Bx cells were derivatives of human hepatoma Hep3B and HepG2 cells, respectively, that stably express the HBX gene and were described previously [44]. Cells were maintained in an atmosphere of 5% CO_2_ in a humidified 37 °C incubator. 

### 4.3. Transient Transfection

The pcDNA6.0-HBx plasmid was constructed by cloning the cDNA product of the HBx gene into the pcDNA6.0 expression vector (Thermo, Waltham, MA, USA) [44]. Transfections were performed using Lipofectamine 2000 or RNAiMAX (Invitrogen). After 48 h, cells were used for PCR, western blot analysis, immunoprecipitation, and migration assays. Oligonucleotides used for PCR and RNA interference were listed in Table S1.

### 4.4. Western Blot Analysis

For total cell lysates, cells were washed with ice-cold phosphate-buffered saline (PBS) one time and lysed in NETN buffer (100 mM NaCl; 0.5 mM EDTA; 20 mM Tris-HCl, pH 8.0; 0.5% [*v*/*v*] Nonidet P-40). Protease inhibitor and phosphatase inhibitor cocktails (Roche, Indianapolis, IN, USA) were included in the NETN buffer. Samples (30 μg) were run on 10% sodium dodecyl sulfate (SDS)–polyacrylamide gels, and the separated proteins were then blotted onto a polyvinylidene fluoride (PVDF) hybridization transfer membrane (PerkinElmer, Branford, CT, USA). The primary antibodies used were anti-Hepatitis B Virus X antigen (Abcam, Burlingame, CA, USA; 1:1000), anti-SHIP2 (Santa Cruz Biotechnology, Dallas, TX, USA; 1:500), anti-SKP2 (Santa Cruz Biotechnology; 1:1000), anti-E-cadherin (GeneTex, Alton Pkwy Irvine, CA, USA; 1:1000), anti-N-cadherin (BD Biosciences, San Jose, CA, USA; 1:1000), anti-vimentin (GeneTex, 1:1000), anti-α-tubulin (Sigma; 1:5000), and anti-β-actin (Sigma-Aldrich Corp, St. Louis, MO, USA; 1:5000). The secondary antibodies used were horseradish peroxidase–conjugated antibodies (Merck Millipore, Danvers, MA, USA). Immunoreactive bands were visualized with the enhanced chemiluminescence detection reagent (GE Healthcare, Piscataway, NJ, USA).

### 4.5. RNA Isolation, Reverse Transcription, PCR, and Real-Time PCR

Total RNA was extracted with TRIzol reagent (Invitrogen). The primers were synthesized by Invitrogen. The cDNA was reverse-transcribed by oligo dT(15) using M-MLV reverse transcriptase (Invitrogen Corp, Carlsbadcity, CA, USA). For SHIP2, HBx, GAPDH, Glut-1, and EMT marker mRNA, 2 μg of total RNA was subjected to reverse transcription with an oligo-dT primer using a reverse transcriptase kit (Invitrogen). Equal amounts of cDNA (2 μL) were subjected to RT-PCR with 30 cycles of amplification. Primer sequences involved in the present study are summarized in Table S1. The RT-PCR products were subjected to 1.2% agarose gel electrophoresis and visualized by ethidium bromide staining. Real-time PCR reactions containing 1 μL cDNA, 1 μL forward and reverse primers, 5 μL 2× SYBR Green (Roche), and 2 μL distilled water were performed with a Roche LightCycler 480 real-time PCR system. The level of mRNA expression was normalized to the level of *GAPDH* mRNA in the same sample.

### 4.6. Immunoprecipitation

Cells were lysed with NETN buffer for 30 min at 4 °C. Cell lysates (1 mg) were mixed with anti-SHIP2 or anti-SKP2 and protein A/G–Sepharose beads and then gently rotated at 4 °C overnight. Immune complexes were then precipitated and subjected to western blotting.

### 4.7. Gene Knockdown with shRNA

Knockdown of genes was performed with specific shRNAs delivered with the lentiviral system from the National RNAi Core Facility (Academia Sinica, Taipei, Taiwan) according to the instruction manual. The shRNA target sequences are summarized in Table S2. For lentivirus transduction, HCC cells were cultured until they reached ~80% confluency, after which they were infected with lentivirus bearing specific shRNAs in growth medium containing 8 μg/mL polybrene for 24 h. Infected HCCs were subcultured for an additional 72 h in growth medium for subsequent experiments.

### 4.8. Cell Viability Assay

Cell viability was determined with the WST-1 (4-[3-(4-iodophenyl)-2-(4-nitrophenyl)-2*H*-5-tetrazolio]-1,3-benzene disulfonate) assay (Roche). After culturing for the indicated time, a one-tenth volume of WST-1 was added. The cells were cultured for 2 h before being harvested, and their absorbance was detected at 450 nm by an ELISA reader. Cell viability was normalized based on the absorbance from the cells without treatment. Cell viability data were obtained from at least three experiments with at least six wells for each concentration in separate 96-well plates.

### 4.9. In Vitro Migration and Invasion Assays

HCC cells were plated in the upper chamber of non-coated Transwell insert (millicell hanging cell culture 24-well insert; pore size, 8 μm; Merck Millipore, Danvers, MA, USA). After incubation for 24 h, the cells that did not migrate through the pores were removed with a cotton swab. Cells on the lower surface of the membrane were fixed and stained with crystal violet. These stained cells, which had migrated through the membrane, were then photographed under a light microscope, then lysed with 33% (*v*/*v*) acetic acid solution and quantified by absorbance measurement (OD570) [45]. The cell invasion assay was similar to the cell migration assay except the Transwell membrane was pre-coated with 1μg/μL Matrigel (BD Biosciences, San Jose, NJ, USA).

### 4.10. Glucose Uptake Assay and Flow Cytometry Analysis 

Glucose uptake in HCC cells was determined using the glucose analog 2-NBDG as described [27]. After infection with SHIP2 shRNA for 72 h, HCC cells were plated at 2 × 10^5^ cells/well in six-well plates and used after a 24 h preincubation (i.e., at subconfluence). The HCC cells were labeled with 50 μM 2-NBDG diluted in a glucose-free medium and incubated for 0.5, 1, and 2 h at 37 °C. Fluorescence images were taken with a Zeiss Axio Observer D1 Microscope (Carl Zeiss, Thornwood, NY, USA).

The 2-NBDG uptake reaction was stopped by removing the incubation medium and washing the cells twice with ice-cold PBS. For each measurement, data from 20,000 single cell events were collected using a FACSCalibur (Becton Dickinson Immunocytometry Systems, San Jose, CA, USA) flow cytometer.

### 4.11. Statistical Analysis

Data analysis was done using Excel (Microsoft) and SigmaPlot 9.0. (Systat Software, San Jose, CA, USA). A two-tailed Student’s *t*-test was used to calculate the statistical significance between the groups. 

## 5. Conclusions

We found a strong inverse relationship between SHIP2 and SKP2 after transfection with HBx plasmid in HCC cells, suggesting that HBx-mediated downregulation of SHIP2 is an important mechanism to induce migration in HCC cell lines through upregulation of SKP2. The results of the present study suggest that reduced expression of SHIP2 might indicate a poor prognosis in HBV-infected HCC patients. These findings may be helpful in developing an effective treatment against HCC.

## Figures and Tables

**Table 1:** Liver cancer tissue array (49 cases/98 cores).

Characteristics	No. (%)
Gender, *n* (%)	
Male	42 (85.7)
Female	7 (14.3)
Age (years)	
Median (range)	50 (24–68)
Stage, *n* (%)	
I	5 (10.2)
II	42 (85.7)
III	2 (4)
HBV infection, *n* (%)	
−	23 (23.5)
+	59 (60.2)
++	16 (16.3)

## Supplementary Materials

The following are available online at https://www.mdpi.com/2072-6694/11/8/1065/s1, Figure S1: Expression of SHIP2 in human HCC tissue without or with HBV infection was examined by IHC analysis of the TMA, which was stained with human SHIP2 antibody, Figure S2: SHIP2 is polyubiquitinated by the E3 ubiquitin ligase SKP2 in HEK293 cells, Figure S3: Knockdown of SHIP2 did not induce resistance to Iressa, Tarceva, Doxorubicin, Sorafenib, and Paclitaxel in Hep3B cells, Figure S4: Raw data of Western blots from Figure 2, Figure S5: Raw data of Western blots from Figure 3, Figure S6: Raw data of Western blots from Figure 5,: Raw data of Western blots from Figure S2. Table S1: Used in This Study, Table S2: Sequences for SHIP2, SKP2, and HBx silencing.
