# Supplementary material for: Downregulation of SHIP2 by Hepatitis B Virus X Promotes the Metastasis and Chemoresistance of Hepatocellular Carcinoma through SKP2
Source: Cancers (Basel). 2019 Jul 27;11(8):1065. doi: 10.3390/cancers11081065 (PMC6721294; doi:10.3390/cancers11081065)
Supplement: Supplementary file 1 [file cancers-11-01065-s001.pdf]

Supplemental Materials: Downregulation of SHIP2 by Hepatitis B Virus X Promotes the Metastasis and Chemoresistance of Hepatocellular Carcinoma through SKP2

Kuo-Jung Su and Yung-Luen Yu

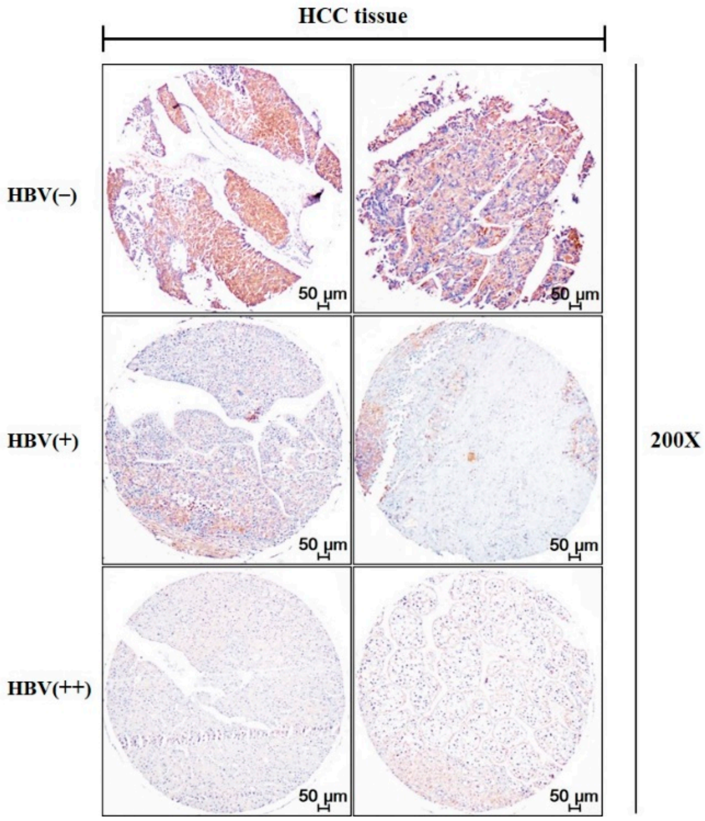

Figure S1. Expression of SHIP2 in human HCC tissue without or with HBV infection was examined by IHC analysis of the TMA, which was stained with human SHIP2 antibody.

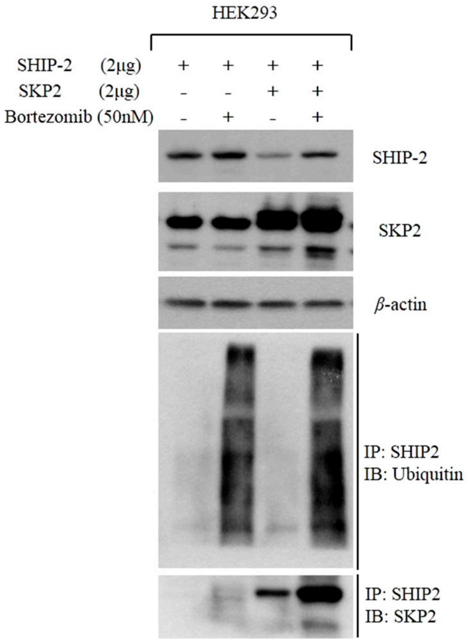

Figure S2. SHIP2 is polyubiquitinated by the E3 ubiquitin ligase SKP2 in HEK293 cells. HEK293 cells were transfected with SKP2 expression vector for 48h. A except that cells were pretreated with bortezomib (50 nM) for 16h prior to cell harvest. Whole protein lysates were prepared followed by immunoprecipitation (IP) with anti-SHIP2. The precipitate was then electrophoresed on a polyacrylamide gel followed by immunoblot (IB) detection using anti-ubiquitin and anti-SKP2.

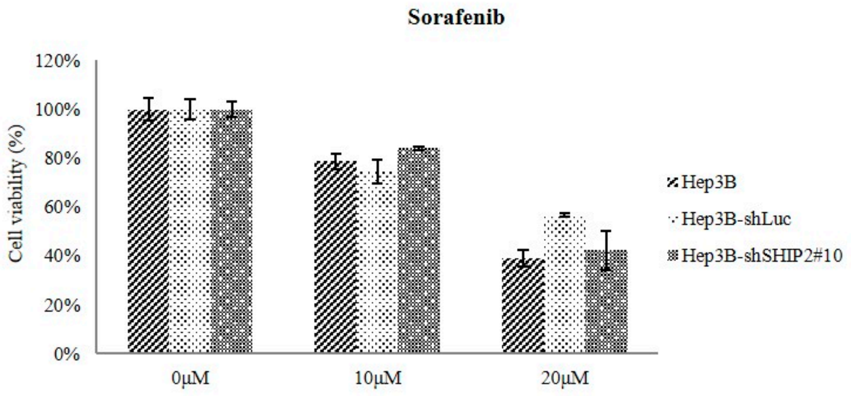
Sorafenib
Cell viability (%)
120%
100%
80%
60%
40%
20%
0%
0μM
10μM
20μM
Hep3B
Hep3B-shLuc
Hep3B-shSHIP2#10

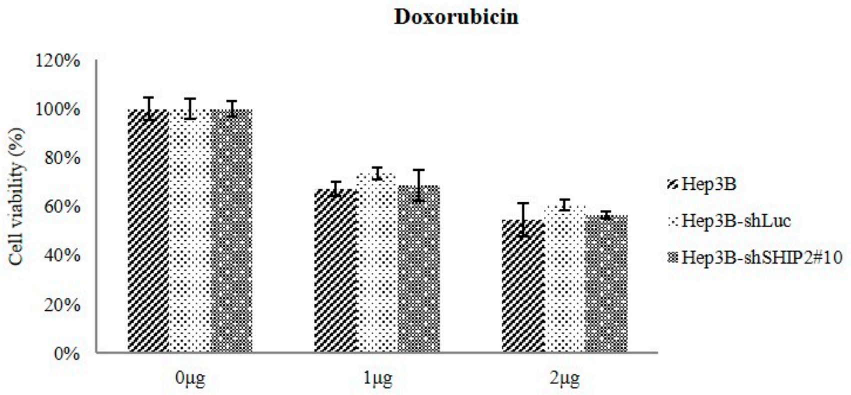
Doxorubicin
Cell viability (%)
120%
100%
80%
60%
40%
20%
0%
0μg
1μg
2μg
Hep3B
Hep3B-shLuc
Hep3B-shSHIP2#10

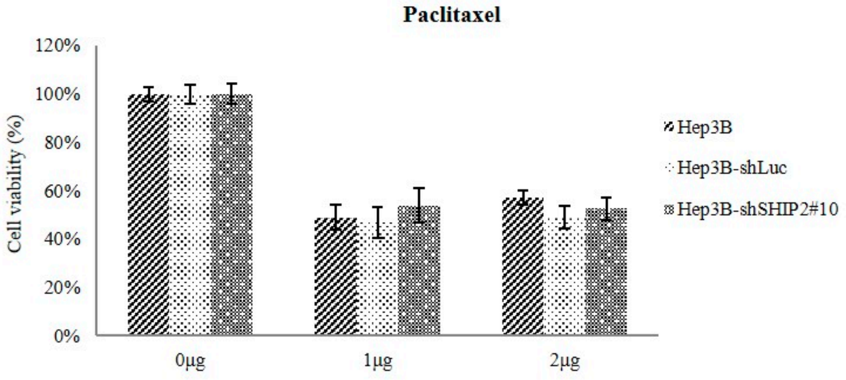
Paclitaxel
Cell viability (%)
120%
100%
80%
60%
40%
20%
0%
0μg
1μg
2μg
Hep3B
Hep3B-shLuc
Hep3B-shSHIP2#10

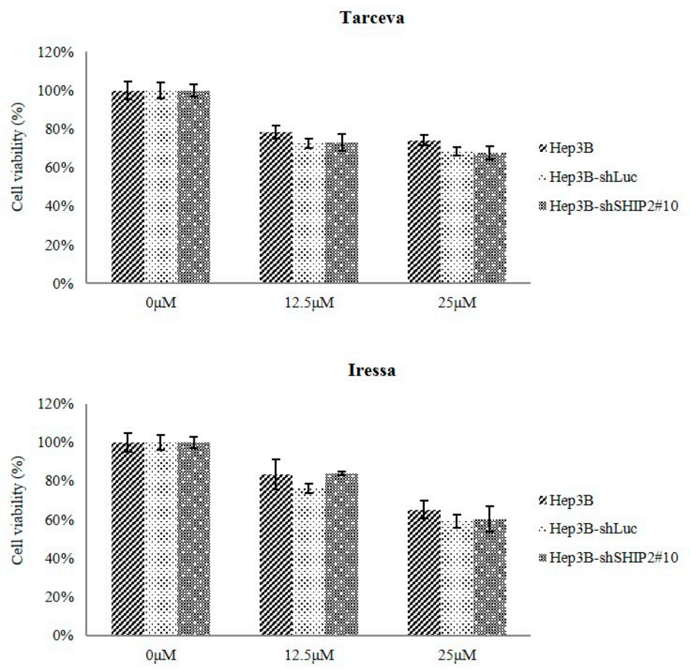

Figure S3. Knockdown of SHIP2 did not induce resistance to Iressa, Tarceva, Doxorubicin, Sorafenib, and Paclitaxel in Hep3B cells. The cytotoxic effects of Iressa, Tarceva, Doxorubicin, Sorafenib, and Paclitaxel in Hep3B cells. The Hep3B cells were plated in 96-well plate after SHIP2 knockdown for 72 h. The cells were treated with indicated doses (12.5 μM and 25 μM) of chemotherapy drugs for 48 h.

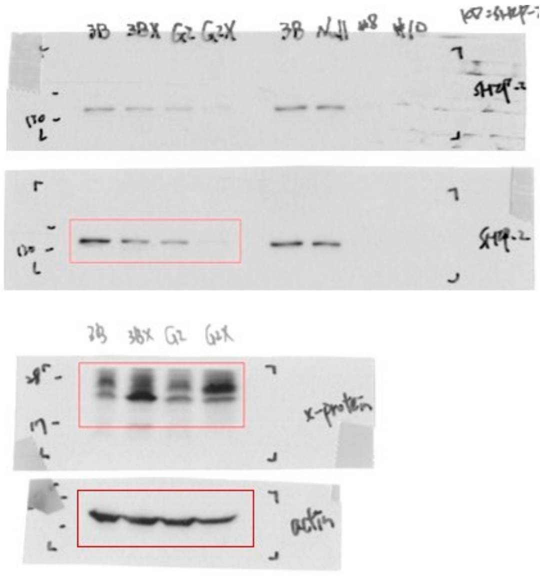

(**A**)

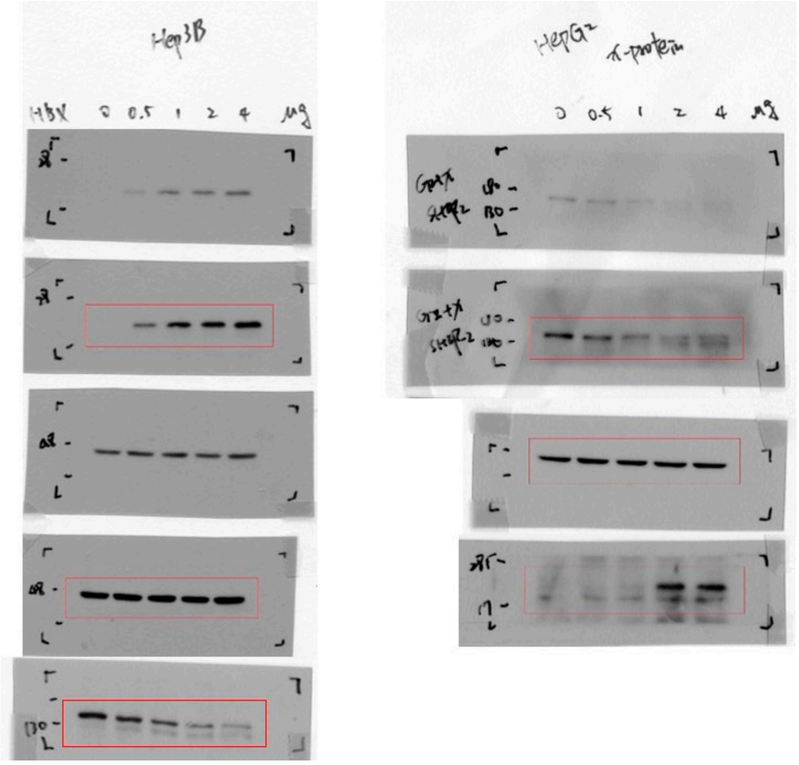
Hep3B
HBX 0 0.5 1 2 4 μg
HepG2 X-protein
0 0.5 1 2 4 μg

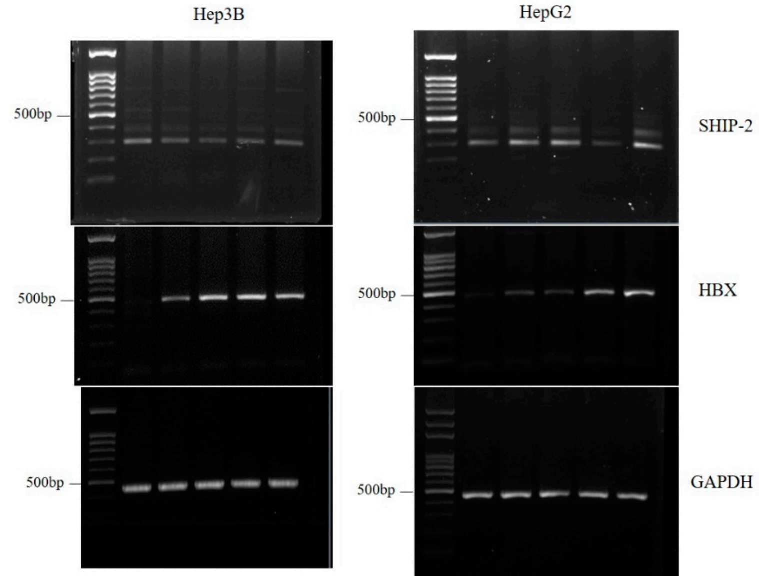
Hep3B
HepG2
500bp
500bp
SHIP-2
500bp
500bp
HBX
500bp
500bp
GAPDH

(**B**)

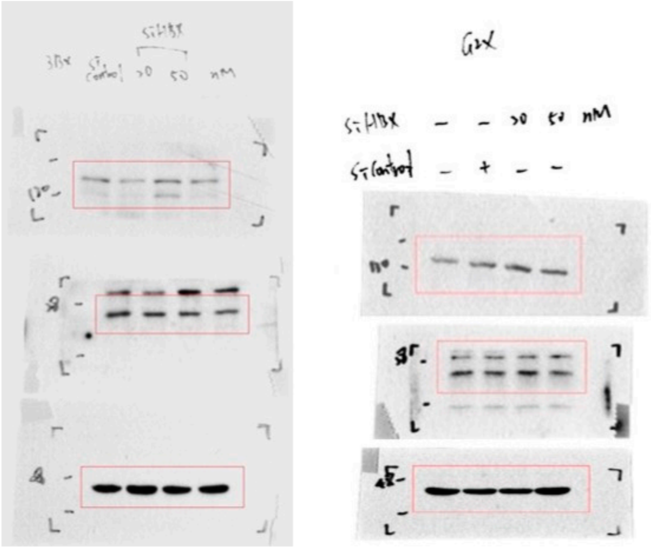

(**C**)

Figure S4. Raw data of Western blots from Figure 2. (**A**) Raw data from Figure 2A. (**B**) Raw data from Figure 2B. (**C**) Raw data from Figure 2C.

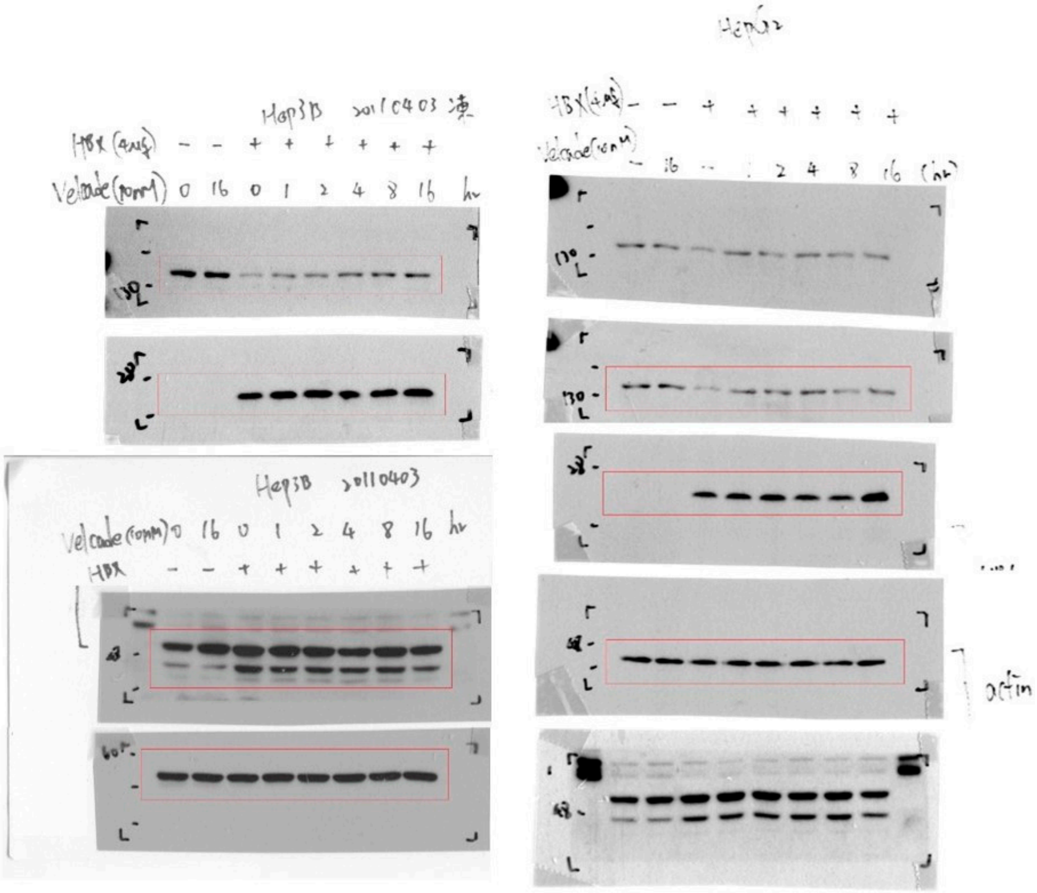

(**A**)

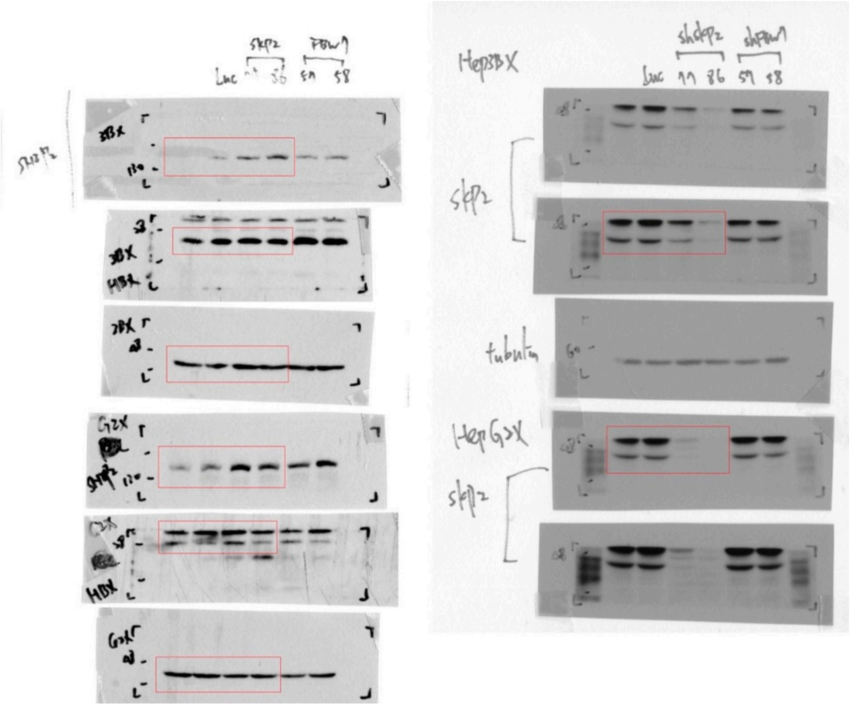

(**B**)

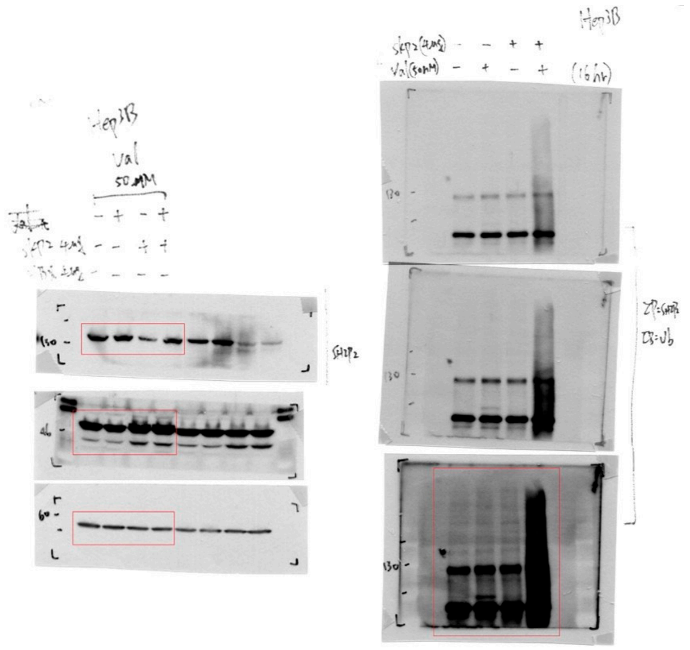

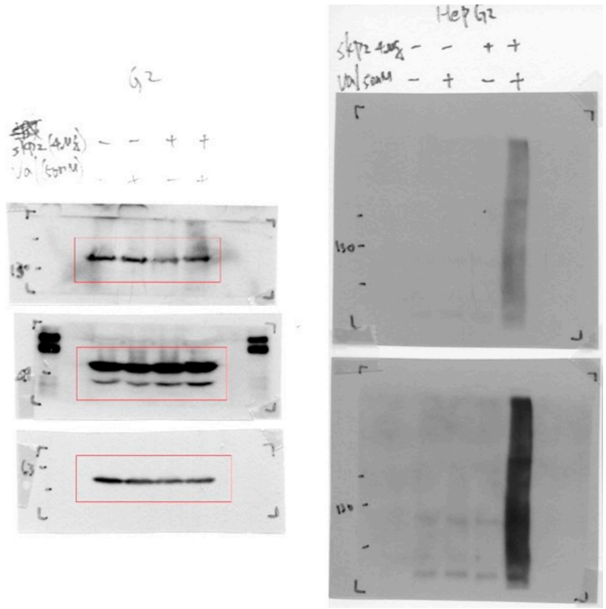

(**C**)

Figure S5. Raw data of Western blots from Figure 3. (**A**) Raw data from Figure 3A. (**B**) Raw data from Figure 3B. (**C**) Raw data from Figure 3C.

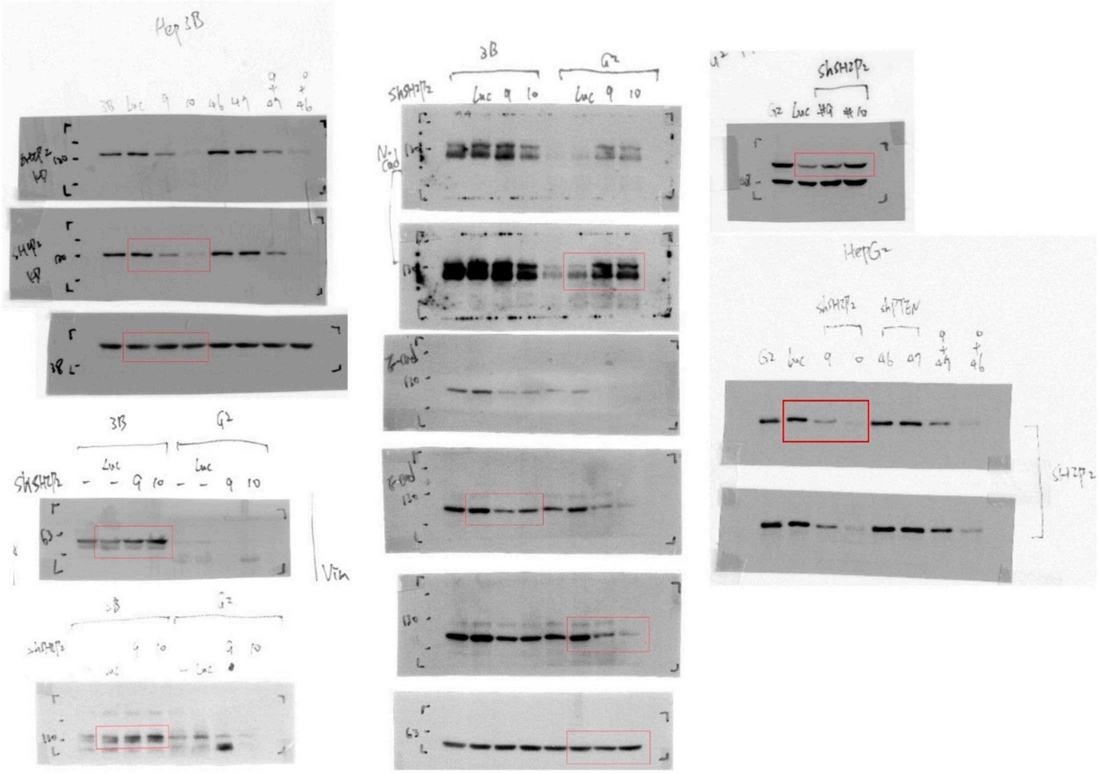

(A)

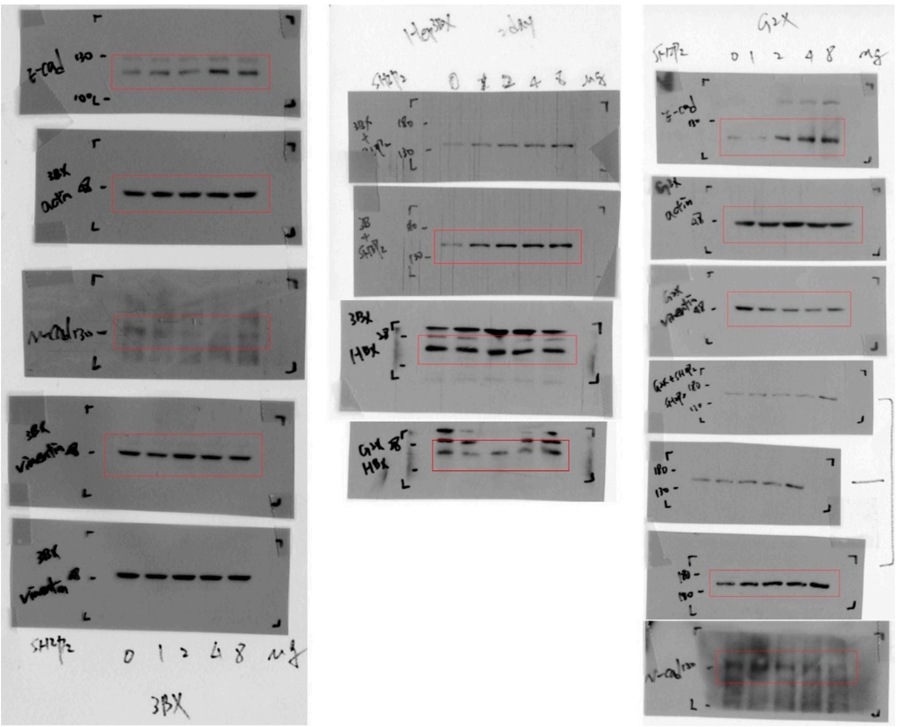

(B)

Figure S6. Raw data of Western blots from Figure 5. (**A**) Raw data from Figure 5A. (**B**) Raw data from Figure 5C.

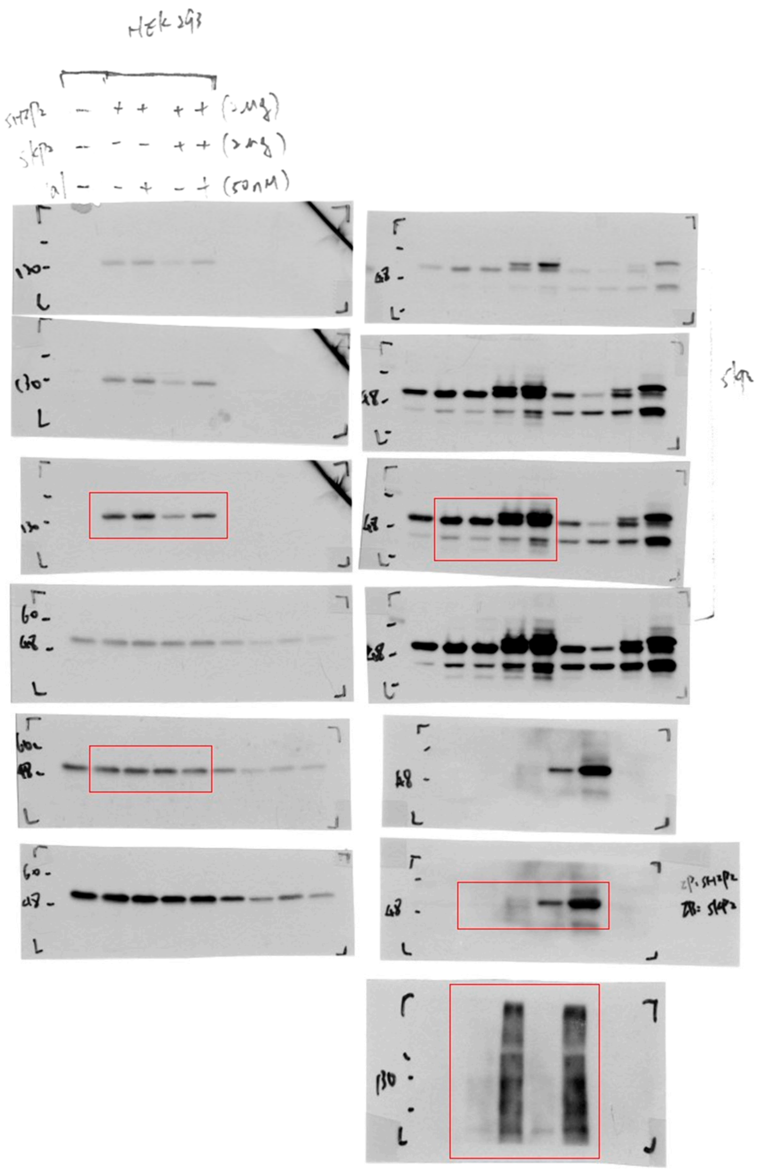

Figure S7. Raw data of Western blots from Figure S2.

Table S1. Used in This Study.

**Target Name**		**Sequence**
E-cadherin	F	5′-GGACCAGGACTTTGACTTGAGC-3′
	R	5′-AATCATAAGGCGGGGCTGTG-3′
N-cadherin	F	5′-CAACGGGGACTGCACAGATG-3′
	R	5′-TGTTTGGCCTGGCGTTCTTT-3′
Vimentin	F	5′-AAAGTGTGGCTGCCAAGAACCTGC-3′
	R	5′-ACTCAGTGGACTCCTGCTTTGCCT-3′
Snail	F	5′-ACCACTATGCCGCGCTCTT-3′
	R	5′-GGTCGTAGGGCTGCTGGAA-3′
GAPDH	F	5′-CCATGGGGAAGGTGAAGGTC-3′
	R	5′-ACATGTAAACCATGTAGTTGAGG-3′
SHIP2	F	5′-GCCGGAGGCTGCACAGTGAG-3′
	R	5′-CCTCAAAGGCCTGCACGGGG-3′
HBx	F	5′-GTTAAGCTTATGGCTGCTAGGCTGTGCTGC-3′
	R	5′-AGACTCGAGCCGGGCAGAGGTGAAAAAGTTGC-3′
Glut-1	F	5′-CGGGCCAAGAGTGTGCTAAA-3′
	R	5′-TGACGATACCGGAGCCAATG-3′

Table S2. Sequences for SHIP2, SKP2, and HBx silencing.

Name	Sequence
shLuc	5′-ATCACAGAATCGTCGTATGCA-3′
shSHIP2#09	5′-CCTGAACTACATCAGCAGGAA-3′
shSHIP2#10	5′-CCGGATTCTGTGGAAATCCTA-3′
shSKP2#77	5′-GATAGTGTCATGCTAAAGAAT-3′
shSKP2#86	5′-GCCTAAGCTAAATCGAGAGAA-3′
HBX siRNA	GGUUAAAGGUCUUUGUAUU
Control siRNA	GAUCAUACGUGCGAUCAGA
